# The legal frameworks that govern fetal surgery in the United Kingdom, European Union, and the United States

**DOI:** 10.1002/pd.5267

**Published:** 2018-05-17

**Authors:** Kevin X. Cao, Alice Booth, Sebastien Ourselin, Anna L. David, Richard Ashcroft

**Affiliations:** ^1^ Department of Urology Great Ormond Street Hospital London UK; ^2^ Leicester De Montfort Law School Leicester UK; ^3^ UCL Centre for Medical Image Computing London UK; ^4^ UCL Institute for Women's Health London UK; ^5^ Department of Development and Regeneration Katholieke Universiteit Leuven Leuven Belgium; ^6^ School of Law Queen Mary University London UK

## Abstract

The specialty of fetal surgery or fetal intervention is one of the most exciting emerging fields of modern medicine. It is made possible by decades of major developments in antenatal imaging, obstetric anaesthesia, fetal medicine, paediatric surgery, and of course by the bold and novel practitioners willing to take new steps to advance the field. Beginning in the 1970s, it has now reached a stage of maturity where there are several established in utero procedures and countless clinical trials and studies to develop more. But what is the legal situation that fetal surgeons find themselves in? What are the rights and legal protections for the fetus and the mother, both of which are arguably the patient? This article will address this question, discussing and summarising the current legal frameworks governing fetal surgery in the jurisdictions of the United Kingdom, European Court of Human Rights, and the United States of America as well as discuss what the future may hold and how researchers and physicians in the specialty can best navigate the legal environment.

## Abstract 2

What's already known about this topic?

- Fetal surgery is entering mainstream clinical care as a specialty that introduces an exciting range of new treatments for mothers and their unborn babies.
- The conflict between maternal autonomy and interests in fetal health is relatively well known. How does fetal surgery affect this issue?

What does this study add?

- Clarifies the legal frameworks that govern fetal surgery in the United Kingdom, European Union, and the United States.
- Examines how fetal surgery may influence the legal position.
- Discusses clinical best practice and how physicians can influence future laws that govern this specialty.

**What's already known about this topic?**

- Fetal surgery is entering mainstream clinical care as a specialty that introduces an exciting range of new treatments for mothers and their unborn babies.
- The conflict between maternal autonomy and interests in fetal health is relatively well‐known. How does fetal surgery affect this issue?

**What does this study add?**

- Clarifies the legal frameworks that govern fetal surgery in the United Kingdom, European Union, and the United States.
- Examines how fetal surgery may influence the legal position.
- Discusses clinical best practice and how physicians can influence future laws that govern this specialty.

## 1 INTRODUCTION

In this article, we examine the parallel evolution of the legal status of the fetus in the United Kingdom, the transnational institutions of Europe and the United States, the growing specialty of fetal surgery (fetal intervention/therapy) and discuss the potential interaction between the two. To date, in all these various legal systems, there has been no direct reference to fetal intervention in legislation or case law. The legal position must therefore be inferred from the positions taken in each legal system with respect to other issues relating to pregnancy and assisted reproduction. We begin by summarising the legal status of the fetus and then assess how the current legal positions impact upon maternal rights and on fetal therapy. Finally, we consider what the future holds for the specialty and how clinicians can shape the way society views the practice both in ethics and in law.

## 2 METHODS

We searched for all relevant case law and statutes in the jurisdictions of the United Kingdom (England and Wales, Scotland, and Northern Ireland), the European Court of Human Rights (ECHR), and the United States on NexisLexis, WestLaw, JustCite, and directly from the databases of the ECHR and the US Library of Congress. We also searched for English language academic articles from those sources and Medline. In all cases, articles were examined for reference to ‘fetal surgery’, consent, abortion rights, forced medical interventions, definitions of legal status, and any other terms deemed relevant to fetal‐maternal therapy.

### 2.1 A brief overview of legal traditions in the three jurisdictions

Laws in England, Wales, Scotland, and Northern Ireland have a complex historical tradition. In England, the ‘Common Law’ system has ancient historic roots in local customary laws and over time through the Medieval and Enlightenment period, increasing introduction of statutory law through parliamentary legislation. A key principle of common law is the importance of judge‐made law or local court decisions. This is case law, which builds over time with judgments setting precedent over future decisions. Statutory law, in some cases, were codifications and, in others, expressions of unwritten common law. This tradition has been applied to Wales since the 16th century and has influence on the jurisdiction of Northern Ireland, in particular in some shared statutory laws, although Northern Ireland has many common law traditions of its own to draw from.^1^ English common law has only partial influence on the legal tradition in Scotland, or Scot's Law, which is a hybrid system of common law with influences from Roman Law that brings it closer to European civil law traditions. All four nations are under the jurisdiction of the Supreme Court of the United Kingdom for matters of civil law and English Acts of Parliament while they are in union. Finally, all four nations of the United Kingdom are also obligatory signatories to the laws of the European Union. This format is largely shared with the United States, which, like other former British colonies, has implemented with adaptations, the English common law tradition. The origin and application of many laws are at state level, with most state legal systems deriving from English common law, although in some cases such as the State of Louisiana, French civil law tradition continues to influence modern legal practice. The main difference in the US law is that the Federal government will enact and promulgate interstate statutes, overriding some existing state legislation.^2^ The European Court of Human Rights (ECHR) is a judicial organ whose primary aim is to enforce implementation of the ‘European Convention on Human Rights’, a contractual statement indicating the Rights and Freedoms under Civil law of European Union citizens. The legal system of the European Union is complex with a historical heritage that derives from the predominantly civil law legal practice of European nations, which trace their roots in national legal traditions as well as Roman law, the codifications of Justinian I and Napoleon, influences from Roman Catholic Canon law, and enlightenment and modern revisions. Civil law critically is based on the principle of ‘codification’ and places greater relative importance on the lawmaker. Importantly, ‘European law’ is characterised by its recognition of the variance among its signatory states, which include both common law and civil law practices. While distinct and supranational, it nevertheless recognises state legal autonomy but also requires participation and implementation of European legal decisions, particularly of the European Courts of Justice and ECHR.^3^ Thus, the jurisdiction of the ECHR is central, governing many nations including currently the United Kingdom.

## 3 EVOLUTION OF FETAL RIGHTS

Historically, the legal status of the fetus has been shaped from the perspective of criminal law governing abortion and (to a lesser extent) the civil law of torts relating to prebirth injury. Through advancing technology, the fetus is increasingly ‘taking on a human form’ in utero before our eyes.^4^ While this focus is evolving, the basis of the law remains the same.

### 3.1 The United Kingdom

The status of the fetus in English law is that it is not a legal person until birth. Abortion or termination of pregnancy was a crime, although rarely if ever prosecuted, in the common law of England, as clarified in the Offences Against the Person Act of 1861 (OAPA, sections 58 and 59).^5^ The Infant Life Preservation Act of 1929 made it a crime to kill or destroy a child who was ‘capable of being born alive’ but was not yet existing independently of the body of the mother, thus closing a loophole in which the act was neither causing a miscarriage (as defined in the OAPA) nor murder or manslaughter (or infanticide), as would be the case if the child was delivered. The case of R v Bourne clarified the law by stating conditions under which the act of causing an abortion could be lawful (the OAPA defining only unlawful abortion as a crime).^6^ In conjunction with the Infant Life Preservation Act, this meant that there was an upper gestational age limit on terminations, which could be deemed lawful. The Abortion Act of 1967,^7^ as amended by the Human Fertilisation and Embryology Act 1990,^8^ formalised and clarified the legal position for lawful abortions and introduced a system of regulation. But the legal history of abortion does not establish any legal rights on the part of the unborn child. It is arguable that the law here does not define a ‘victim’, rather it defines a class of wrongful acts and focusses on the acts and the agent.^9^ Of note, this does not apply in Northern Ireland, where the Abortion Act 1967 has not been applied.

This sets out the main protection of fetal rights in the UK law; indeed, it was stated in *Paton v British Pregnancy Advisory Service* that ‘the fetus cannot, in English law…have any right of its own…until it is born and has a separate existence from the mother’.^10^ At this point, however, the legal position becomes less clear; forced caesarean section cases whereby physicians have successfully applied for court‐mandated caesarean delivery in cases of impending fetal harm,^11^, ^12^, ^13^, ^14^ have weakened this stance as they represent instances of doctors, as argued in *R v S*, acting primarily for the benefit of a fetus, which by law has no legal rights.^15^ A recent test case exploring the legality of compensating a child suffering Fetal Alcohol Syndrome was ruled in line with the stance in *Paton*;^16^ a child cannot sue its mother for in utero harm. This however is a civil law case. The law here is complex, but the priority of the mother in ethics and law remain central.

### 3.2 Member states of the Council of Europe

This overarching jurisdiction covers signatory states to the European Convention on Human Rights (ECHR, 1953), which sets out rights afforded to all legal persons including the Article 2—Right to life.^17^ Whether this article applies to the fetus was tested in *Vo v France*.^18^ In this case, physicians of a pregnant French woman, Mrs Vo, attempted to remove a non‐existent intrauterine device due to a mix‐up with a similarly named patient, resulting in miscarriage. A claim was brought forward by her lawyers stating the lack of criminal charges for loss of fetal life was incompatible with the state duty to protect the right to life. The ECHR, under great internal pressure, ultimately avoided clarifying the position of the fetus and how much protection it should be given by refusing to pass judgment.

While many European states share the Napoleonic code as the foundation of their legal systems, the actual legislation is individual to each nation. The legislation of traditionally Catholic countries is generally stricter on abortion than their more secular cousins, which allow greater access.^19^ This difference in attitudes is one source of the ECHR's reticence in ruling on the fetus; individual member states have a duty to implement legislation in line with the decisions of the Court and a duty to change their law if not compatible. Thus, ECHR decisions demand approval from the social, religious, and political feelings of its member nations; as international law is founded on compromise, when one cannot be found, the best solution to preserve the overall jurisdiction is sometimes to stay silent on the matter.

### 3.3 The United States

The United States has seemingly taken a more proactive approach in stating its position regarding the fetus. Abortion laws stem from the *Roe v Wade* interpretation of the 14th Amendment to the Bill of Rights,^20^ which provides a right to abortion balanced against the State's interest in prenatal life, a partial, not absolute, right to abortion.

Previously, a civil action for fetal harm was not possible as legal rights are conferred with ‘personhood’ at birth; however, legislation has shifted perspective so that personhood can be found in the womb, as evidenced by the Unborn Victims of Violence Act.^21^ Although this only applies to violent crimes, it represents a willingness to award rights and protections before birth where previously none existed.

Cases of intervention to preserve fetal life have also been decided, one dramatic example being the case of Marlise Muñoz concerning whether a pregnant comatose woman should be kept on life support until her fetus reached viability.^22^ The 1990s and 2000s featured cases involving women being charged with, among other examples, wilfully endangering a child in *Sherriff v. Encoe*,^23^ criminal abuse in *Commonwealth v. Welch*,^24^ and being sentenced to prison in *Commonwealth v. Kemp* for consuming illicit drugs during pregnancy.^25^ Many of these decisions were criticised and reversed on appeal.^26^ The number of cases of this nature has reduced over the past decade perhaps indicating that this trend is reversing and the courts are reasserting the importance of maternal rights.

State interpretation of federal statutes on this emotionally charged issue is understandably subjective. The US courts appear to sit in a middle ground between the United Kingdom and ECHR as the former has seemingly ruled out fetal rights while the latter refuses to state its position. The United States has attempted to find a method of implementing both fetal and maternal rights with some success and failure, resulting in an ambiguous position; earlier cases imply favour to fetal rights, and subsequent events suggest maternal rights have returned to primacy.

## 4 EFFECT ON MATERNAL RIGHTS

A key issue regarding fetal rights is the difficulty in applying legal personhood rights to the fetus and mother when one is not only part of the other's body but ‘interconnected… in [so] many intricate and intimate ways’.^27^ This is known as the ‘fetal‐maternal conflict’, when acting for one diminishes the rights of the other.

### 4.1 The United Kingdom

The effect of fetal rights on maternal rights in the United Kingdom has been limited; forced caesarean section cases are the best evidence of the conflict. Almost all the UK cases have revolved around doctor‐patient disputes at the time of childbirth rather than court‐imposed sanctions arising from physician concerns earlier in pregnancy about fetal well‐being.

The court sets out several guiding principles in the case of *Re MB*, a landmark case involving a woman with needle phobia refusing caesarean section. Those principles aimed at reducing the instance of these cases going to court, denote that in moments of fear or panic, mental capacity may be lost and the physician may act in the best interest of the patient and in this case the fetus.^10^ Unfortunately, fear and panic are not difficult to find in many births.^11^ Reassuringly for proponents of maternal autonomy, most of these decisions were ultimately reversed on appeal.

### 4.2 Member states of the Council of Europe

The ECHR has few instances of fetal‐maternal conflict, but there are numerous cases on reproductive rights and autonomy that reveal the extent to which the Court is willing to protect the mother. The ECHR has repeatedly ruled in favour of women undergoing forced sterilisation such as in *K.H. and others v. Slovakia*,^28^ finding a breach of the women's ‘Article 8—right to respect for private and family life.’ The most relevant case concerns consent to sterilisation given without full understanding during labour in *VC v Slovakia*;^29^ here, the court found in favour of the woman because her understanding was not secured. As there is little evidence on the Council's position regarding the fetal‐maternal conflict, reaching a conclusion is difficult, but these cases hint at a pro‐maternal autonomy lean.

What *VC v Slovakia* also demonstrates is the focus of the Court on respecting and protecting the cultural and religious views of each member nation; a prominent factor in this decision was the social exclusion the mother endured from the Roma community after sterilisation. Europe has many cultural and religious differences, which impact upon public opinion and legislation on the relationship between mother and fetus, an example being the greater restriction on maternal autonomy through restriction of access to abortion in more traditionally religious states.^30^, ^31^

### 4.3 The United States

The effect of the fetus on maternal rights can be seen most clearly in the law of the United States, which has the largest number of cases of maternal rights being infringed in favour of the fetus; state legislation ranges from classifying pregnant recreational drug use as assault,^32^, ^33^, ^34^, ^35^, ^36^, ^37^, ^38^, ^39^ imprisoning mothers to prevent recreational drug use,^40^ to making the fetus a ward of court.^41^

What these cases represent is the conflict in the US legal system between various pressure groups, public opinion, and legislation; while some cases show a preference to protecting the fetus sometimes at the cost of maternal rights, most of these cases were ultimately repealed. Legislation such as the Unborn Victims of Violence Act,^21^ which although does not affect maternal rights directly, does show a willingness in the US law to transfer legal personhood into the womb, the implications of which may affect maternal rights.

## 5 THE LEGAL POSITION ON FETAL INTERVENTION

Fetal intervention has advanced tremendously since the 1970s with many new therapies translating from research into clinical practice as research trials demonstrate efficacy.^42^, ^43^, ^44^ Fetal intervention undertaken for research is protected legally by robust research ethical coda, but as the specialty becomes established outside of research, it will attract observation and interest from the law.

A pivotal question is how treatment of the fetus affects the rights proscribed to it. ‘Patienthood’ is a status that awards an individual the right to medical treatment, and this comes from the individual's legal ‘personhood’—usually awarded after birth. The question is, does this also have the reverse effect? Does treating the fetus as a patient mean that personhood and its accompanying rights will be awarded in the womb?

### 5.1 The United Kingdom

In the United Kingdom, this is a grey area, as performing medical treatment on a person without consent is to commit assault.^45^ As the intervention is for the benefit of the fetus rather than the mother, this appears in contradiction with the law. Currently, there is little impetus to legislate on the matter and no case has been brought before the courts so judgment has simply not been passed; until this is resolved, fetal intervention exists in a grey area that for now is considered legal in so much as the law protects and enshrines maternal rights of autonomy over their bodies.

Fetal intervention is currently governed by existing medical negligence frameworks with the fetus treated as an organ of the mother. This position satisfies the legality question but does not adequately consider the psychosocial burden this places on the mother. There is considerable personal, familial, and societal pressure on pregnant women to act selflessly for their unborn child; she may feel she ‘has no choice’.^46^, ^47^ When assessing a prenatal therapy, many pregnant women consider their fetus as a baby, even if they have not attained a ‘viable’ gestational age.^48^ As fetal intervention moves increasingly into the mainstream, placing safeguards to prevent diminution of maternal autonomy may protect mothers in the long run. Furthermore, the fetus's lack of rights prevents its classification as a patient, so it is not supported by the personhood/patienthood structure. However, this is not necessarily relevant; to deduce the status of a being from the treatment given to them is to forget the ‘fundamental[ly] different nature of law and medicine’.^49^ Although medical practice can help understand these issues, ultimately, it is for the law to decide the status of the fetus.

What is more relevant is that fetal surgery is now established as an effective and successful medical therapy that has brought about an exciting new array of treatments for the fetus. Medical law has throughout history had to adapt with evolving practices, so it seems unlikely that the law will not do so here.

### 5.2 Member states of the Council of Europe

The position regarding the member states of the European Council is much less clear as each is governed by its own domestic legislation. As the law stands currently, the legality of fetal intervention is not explicitly confirmed, as the European Court will not state whether the Convention applies to the fetus. If the Convention were to apply, it is likely that the fetus's right to life would carry a corresponding right to medical treatment making intervention legal, and the reverse would be true if the Convention did not apply.

Were the Court to pass a ruling either way on the Convention's applicability the impact would be far reaching: All domestic legislation that does not treat the fetus as a full rights‐holder would need to be reviewed if not abandoned. This seems unlikely as the Court has found nations culpable for not offering access to abortion.^31^, ^50^ It seems most likely that the Court will simply continue to abstain from judgment to avoid the disruption that such a decision would bring.

### 5.3 The United States

As with the other jurisdictions, the United States has no current legal position on fetal intervention but seems closer to arriving at one. Steps have been taken in protecting the fetus legally, demonstrating that the United States has declared its legal interest in fetal life. This means that the framework for legal intervention has already been laid in embryonic form at least.

This does not guarantee that intervention will be formally legalised; the influence of pressure groups on both the Pro‐life and Pro‐choice side of the debate is considerable and has had an impact on legislation in the past. Moreover, the reversal of many of the prominent fetal‐maternal conflict cases has made the US position equally ambiguous. In this area, cultural and public opinion plays a great role, so if the trend has indeed shifted to favour maternal rights, then perhaps regulating fetal intervention will be more challenging.

## 6 FUTURE TRENDS FOR THE SPECIALTY

How the practice of fetal intervention could affect future laws regulating the relationship between mother and fetus is under debate. A central question is whether ascribing patienthood to the fetus also confers personhood in the womb. This would result in two individual rights‐holders occupying the same body, an eventuality in which the instruments that award these rights are not prepared for at present. There have been sensational concerns that awarding the fetus rights could reduce women to ‘ambulatory wombs’,^51^ although this contrasts with other views that interventions can extend maternal choice and autonomy.^46^ Removing the opportunity to undergo therapy for the benefit of the fetus closes off valuable treatment routes that may improve fetal outcomes and indirectly maternal and familial psychosocial well‐being. It is often forgotten in these debates that the psychosocial burden of raising a child is mainly a private one,^52^ so limiting choice may have far‐reaching consequences for fetus and parents.

There is concern about the effect on abortion legislation, as the legal basis of abortion relies on the fetus's lack of personhood. It is unlikely to prevent all legal termination but may reduce availability, especially late gestation abortions if fetal therapy becomes an option.

Another intriguing prospect is that of the artificial placenta, a potential technical advance whereby a fetus may be delivered from the mother and subsequently supported outside the womb, completing its gestational development without biologic support of the mother.^53^ In this scenario, fetal surgery could be performed without impacting maternal health. It is difficult to predict how the law will respond to this scenario, but there are numerous potential positions the law could take, all of which may lead to ethical and social questions. There is no doubt that there is the clinical desire for these technologies to develop as a solution to spontaneous or iatrogenic preterm birth, which already poses a great burden on society.

As fetal therapy advances, legislators will be forced to reach a decision on the position of the fetus and the rights that it should be accorded. For all three jurisdictions, this will be difficult, in finding a resolution that sensitively weighs up differences in cultural values and binds people on such an emotive issue as the treatment of the fetus.

What is also increasingly clear is that the constantly shifting world of politics has a great impact on the frameworks of each nation's laws. Changing political environments and the results of popular movements can have the effect of altering the social perspective on how new laws should be written or old laws changed. In the case of the United Kingdom, for example, the jurisdictions themselves will change, likely to reaffirm the country's native laws rather than those of the ECHR. In today's state of play, it is as important as ever for physicians to recognise their role in shaping how their specialty is regulated by the law.

## 7 THE ROLE OF FETAL THERAPY PRACTITIONERS

While clinicians are unable to directly dictate legislation, their actions drive future decisions, and it is largely for clinicians to decide how this effect will culminate, how and when clinical services are offered, and the detail of ethical guidelines in this field. As fetal intervention is driven by research endeavours around the world, the effect that physicians have on the medical, social, and legal implications of their study is great. The translation of research could reinforce the specialty with a solid *framework of principles* that will shape how future laws will appear.

The pathway for innovation in fetal therapy was defined many years ago by the International Fetal Medicine and Surgery Society (IFMSS). It is suggested that fetal medicine and surgery practitioners collaborate to produce acceptable indications and outcomes for their practice while respecting research equipoise. Cooperative agreement by physicians in the form of a framework is in the long run more readily adopted by legislators (see Table 1).

**Table 1:** International Fetal Medicine and Surgery Society (IFMSS) mission statement

International Fetal Medicine and Surgery Society mission statement
1. To promote and encourage the development and advancement of the field of fetal diagnosis and therapy 2. To advance the cause of education and scientific research relating to the field of fetal diagnosis and therapy or other reasonably related medical or scientific pursuits 3. To promote the establishment of a mutually beneficial relationship among its members

Medical treatment often advances through the courage to experiment with new techniques, technology, and practice beyond established standards. This of course should be encouraged, and innovation must be supported in the fetal medicine and surgery community. A good standard to follow would be to seek institutional ethical approval before undertaking a new practice. The outcomes of new practice should be presented internationally at established academic meetings at the earliest possible opportunity for peer review. It is also incredibly important to disseminate information to and involve relevant patient and public interest groups, particularly those that advocate for mothers and for patients living with the conditions that new fetal interventions are designed to treat. It is as important to report on negative outcomes, as well as successes. The international community of practitioners, armed with a solid body of evidence, is then able to derive a consensus on successful treatments and to agree on disengaging in potentially dangerous practice. International committees should also actively direct treatments with the greatest potential into properly conducted clinical trials to allow the evaluation of the innovation against current gold standard clinical practice, or where no treatment is available, against current untreated clinical outcomes. These often require registries of natural history data to be set up, to compare with treated cohorts in clinical trials, examples of these are emerging in the fetal medicine community.^54^ This is especially important as the specialty is still small and patient numbers limited relative to the greater practice of medicine. This approach, taken as the individual practitioner and a member of the growing collective body of fetal physicians, supports the ‘frontiersman attitude’ in a safe and legally defensible manner.

Ultimately, the law, like the medical profession, wishes to see advances occur and for patients to have greater choice and better care. Having learned historical lessons from other specialties and seen its own rise from obscurity to prominence, fetal surgery should now take stock of these lessons and understand the nature of law. What the law would like to see is unity in opinion, whether it is a firm stance on a treatment or the clear indication that the scientific community is heading in that direction.

## CONFLICTS OF INTEREST

None declared

## FUNDING SOURCES

The concepts in this work were discussed at the Patient Public Engagement Group of the GIFT‐Surg project http://www.gift-surg.ac.uk), which is supported by an Innovative Engineering for Health award by the Wellcome Trust (WT101957) and the Engineering and Physical Sciences Research Council (EPSRC) (NS/A000027/1). A.L.D. is supported by the UCL/UCLH NIHR Comprehensive Biomedical Research Centre.

## LEGAL REFERENCE KEY

EWHC QB/KB: : High Court of England and Wales Queen's/King's Bench Division
EWHC Fam: : High Court of England and Wales Family Division
EWCA Crim: : High Court of England and Wales Court of Appeal Criminal Division
UKHL: : United Kingdom House of Lords
ECHR: : Council of Europe, European Court of Human Rights
